# Edaravone Enhances Brain-Derived Neurotrophic Factor Production in the Ischemic Mouse Brain

**DOI:** 10.3390/ph8020176

**Published:** 2015-04-02

**Authors:** Satoshi Okuyama, Mayu Morita, Atsushi Sawamoto, Tsukasa Terugo, Mitsunari Nakajima, Yoshiko Furukawa

**Affiliations:** Department of Pharmaceutical Pharmacology, College of Pharmaceutical Sciences, Matsuyama University, 4-2 Bunkyo-cho, Matsuyama, Ehime 790-8578, Japan; E-Mails: lmv_mayu_laulea@yahoo.co.jp (M.M.); 46140018@cc.matsuyama-u.ac.jp (A.S.); 66140105@cc.matsuyama-u.ac.jp (T.T.); mnakajim@cc.matsuyama-u.ac.jp (M.N.); furukawa@cc.matsuyama-u.ac.jp (Y.F.)

**Keywords:** edaravone, global cerebral ischemia, hippocampus, BDNF, neurogenesis, CaMK II

## Abstract

Edaravone, a clinical drug used to treat strokes, protects against neuronal cell death and memory loss in the ischemic brains of animal models through its antioxidant activity. In the present study, we subcutaneously administrated edaravone to mice (3 mg/kg/day) for three days immediately after bilateral common carotid artery occlusion, and revealed through an immunohistochemical analysis that edaravone (1) accelerated increases in the production of brain-derived neurotrophic factor (BDNF) in the hippocampus; (2) increased the number of doublecortin-positive neuronal precursor cells in the dentate gyrus subgranular zone; and (3) suppressed the ischemia-induced inactivation of calcium-calmodulin-dependent protein kinase II in the hippocampus. We also revealed through a Western blotting analysis that edaravone (4) induced the phosphorylation of cAMP response element-binding (CREB), a transcription factor that regulates BDNF gene expression; and (5) induced the phosphorylation of extracellular signal-regulated kinases 1/2, an upstream signal factor of CREB. These results suggest that the neuroprotective effects of edaravone following brain ischemia were mediated not only by the elimination of oxidative stress, but also by the induction of BDNF production.

## 1. Introduction

Brain ischemia poses a serious risk to human health because it induces neuronal cell death in the brain, which is accompanied by physical disabilities and/or cognitive impairments [1,2]. The formation of reactive oxygen and nitrogen species that culminate in oxidative stress has been shown to induce neuronal cell death during ischemia/reperfusion [3,4]. Edaravone, a free radical scavenger, is widely used in Japan for acute ischemic strokes [5,6] as one of the limited therapeutic drugs for ischemic insults [7,8]. On the other hand, recent studies reported that brain-derived neurotrophic factor (BDNF), a representative neurotrophic factor in the brain, played a neuroprotective role following cerebral ischemia injuries, and evidence is accumulating to show that BDNF is important for ischemic brain therapy [9,10]. In an *in vitro* experiment, Wang *et al.*, showed that edaravone exhibited its neuroprotective roles by enhancing the expression of BDNF and Bcl-2, suppressing caspase-3 activity, and promoting the activation of extracellular signal-regulated kinases 1/2 (ERK1/2) in cultured neurons [11]. Almeida *et al.* demonstrated that the activation of ERK1/2 was a critical step for stimulating the synthesis of BDNF [12].

We previously reported that 3,5,6,7,8,3',4'-heptamethoxyflavone (HMF), a citrus flavonoid, increased the expression of BDNF and protected neurons from cell death in the hippocampus of ischemic brains, and that most BDNF-positive cells were also stained with glial fibrillary acidic protein (GFAP, one of the major intermediate filament proteins of mature astrocytes) [13,14]. We also showed that HMF induced the activation (=phosphorylation) of ERK1/2 in the hippocampus following ischemia [13]. These findings prompted us to investigate whether edaravone possessed the ability to stimulate the synthesis of BDNF via astrocytes in ischemic brains *in vivo*, similar to HMF.

## 2. Experimental Section 

### 2.1. Animals

C57BL/6 strain mice (nine-week-old males) were purchased from Japan SLC (Hamamatsu, Japan). The mice in all groups were kept at 23 ± 1 °C on a 12-h light/dark cycle (light on 8:00–20:00). During the experimental period, tap water and feed were freely available.

All animal experiments were carried out in accordance with the Declaration of Helsinki and the Guidelines for Animal Experimentation prepared by the Animal Care and Use Committee of Matsuyama University (approved in 02/09/2009, protocol No. 09-002).

### 2.2. Global Cerebral Ischemia Procedure

Mice were anesthetized with 1.0%–2.0% isoflurane and subjected to bilateral common carotid artery occlusion (2-vessel occlusion: 2VO) as previously reported [13,14,15]. To induce transient global ischemia, micro-aneurysm clips (#14120, 30 g pressure; World Precision Instruments, Sarasota, FL, USA) were applied for 12 min to occlude arteries. Sham control animals (Sham group) received the same surgical treatment without arterial occlusion. Core body temperature was maintained during surgery with a rectal probe and heated blanket (37.0 ± 0.5 °C). Brain temperature was monitored with a tympanic membrane probe into the ear and maintained at 36.5 ± 0.2 °C. Mice were excluded if their brain temperatures and core temperatures were out of range of the criteria set during ischemic surgery because hypo- and hyperthermia have been shown to attenuate ischemic damage in the brain [16,17]. After surgery, all mice were placed in a recovery cage under a heat lamp and had free access to drinking water.

### 2.3. Drug Treatment

Edaravone (3-methy-1-phenyl-2-pyrazolin-5-one) was purchased from Toronto Research Chemicals Inc. (Toronto, ON, Canada) and dissolved in DMSO/PEG300 (1:1) solution. Edaravone was subcutaneously administrated (3 mg/kg/day) to 2VO-treated mice (2VO+EV group; *n* = 9) using an Alzet osmotic pump (1003D, 1.0 μL/h; DURECT Corporation, Cupertino, CA, USA). In the two other groups (Sham group and 2VO group; *n* = 9, respectively), vehicles (DMSO/PEG300) were subcutaneously administrated using an Alzet osmotic pump. Osmotic pump implantation was performed immediately following ischemic surgery and was continued for three days.

### 2.4. Immunofluorescence for Confocal Microscopy

Three days after surgery, mice were transcardially perfused with ice-cold PBS. The brain was removed and half of it was postfixed as described in our previous study [13,14,15]. Thirty-micrometer-thick sagittal sections were incubated with the following primary antibodies; a rabbit anti-BDNF antibody (1:150; Epitomics, Burlingame, CA, USA), mouse anti-GFAP antibody (1:200; Sigma-Aldrich, St. Louis, MO, USA), goat anti-doublecortin (DCX) antibody (1:50; Santa Cruz Biotechnology, Santa Cruz, CA, USA), rabbit anti-phospho calcium-calmodulin-dependent protein kinase II (p-Thr286 CaMK II) antibody (1:500; Sigma-Aldrich), mouse anti-neuronal nuclei (NeuN) antibody (1:300; Millipore, Billerica, MA, USA), and rabbit anti-inducible nitric oxide synthase (iNOS) antibody (1:50; Abcam, Cambridge, UK). Alexa Fluor 488 goat anti-rabbit IgG (H+L) (1:300; Invitrogen, Carlsbad, CA, USA), Alexa Fluor 488 donkey anti-goat IgG (H+L) (1:300), Alexa Fluor 568 goat anti-rabbit IgG (H+L) (1:300), and Alexa Fluor 568 goat anti-mouse IgG (H+L) (1:300) were used as secondary antibodies. A mounting medium with DAPI was used (Vectashield; Vector Laboratories, Burlingame, CA, USA), and images were captured with a confocal fluorescence microscopy system (LSM510; Zeiss, Oberkochen, Germany). Positive signals were quantified using “analyze particle” tool in Image J software (NIH, Bethesda, MD, USA). Using the tool, positive signals were counted over a criteria size.

### 2.5. Western Blot Analysis

The hippocampal region was dissected out from the other half of the brain, weighed, and homogenized in 10 volumes of RIPA buffer (20 mM Tris-HCl, pH 7.5, 150 mM NaCl, 0.1% SDS, 1% sodium deoxycholate, 1% NP-40, 2 mM EDTA, and a protease inhibitor cocktail (Roche, Mannheim, Germany)). Lysates were centrifuged at 20,000 × g at 4 °C for 30 min and supernatant solutions were collected as protein extracts. Equal amounts of protein (25 μg) were separated on 10% SDS polyacrylamide gels and electroblotted onto an Immuno-BlotTM PVDF Membrane (Bio-Rad, Hercules, CA, USA) as previously described [13]. The primary antibodies used in the immunoblotting analysis were a rabbit antibody against MAPK 1/2 (ERK1/2), which recognizes 44-kDa MAPK1/ERK1 and 42-kDa MAPK2/ERK2 (Millipore, Billerica, MA, USA); a rabbit antibody against phospho-p44/42 MAPK (Thr202/Tyr204), which recognizes phosphorylated ERK1/2 (pERK1/2; Cell Signaling, Woburn, MA, USA); and rabbit antibodies against CREB (Cell Signaling) and phosphorylated CREB (Ser133; Cell Signaling). The secondary antibody was horseradish peroxidase-linked anti-rabbit IgG (Cell Signaling). Immunoreactive bands were visualized by ECL-plus (GE Healthcare, Chalfont St. Giles, UK) and their intensities were measured using a LAS-3000 imaging system (Fujifilm, Tokyo, Japan).

### 2.6. Statistical Analysis

Data for individual groups are expressed as means ± SD. Data were analyzed by unpaired t-test (Prism 6; GraphPad Software, La Jolla, CA, USA). A value of *P* < 0.05 was considered significant.

## 3. Results and Discussion

**Figure 1:**
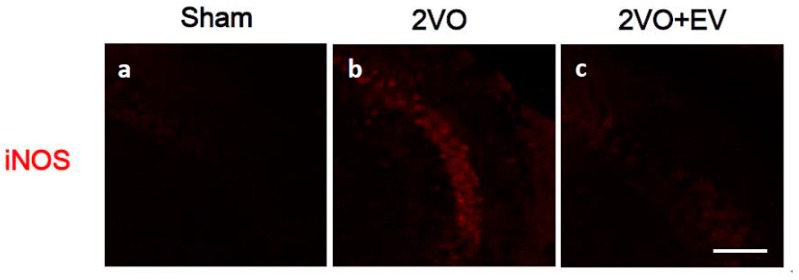
Effects of edaravone on the expression of iNOS immunoreactivity in the mouse hippocampal CA3 region; (**a**) Sham, (**b**) 2VO and (**c**) 2VO + EV. Sagittal sections three days after 2VO surgery were stained with the anti-iNOS antibody (red). Representative immunohistochemical staining were shown from four images per each group. The scale bar shows 100 μm.

**Figure 2:**
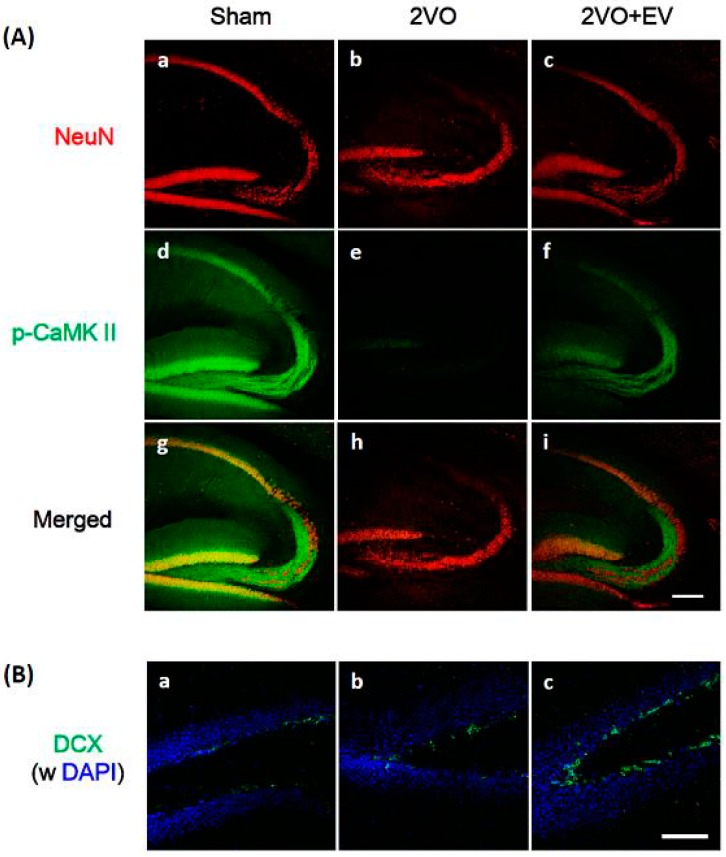
Effects of edaravone on the expression of the immunoreactivity of NeuN and p-CaMK II in the hippocampus (**A**) and DCX in the dentate gyrus (**B**) of the mouse hippocampus. Sagittal sections three days after 2VO surgery were stained with specific antibodies against NeuN (red), p-CaMK II (green) or DCX (green). Nuclei were stained with DAPI (blue). Representative immunohistochemical stainings were shown from eight images per each group. The scale bars of A and B show 200 μm and 100 μm, respectively.

Ischemia/reperfusion leads to neuronal cell death due to the induction of glutamate excitotoxicity, membrane depolarization, increases in cellular calcium levels, and the formation of reactive oxygen and nitrogen species that culminate in oxidative stress [3,4]. In order to confirm edaravone’s scavenging properties in the present animal model, we evaluated its effects on the expression of iNOS in the CA3 region of the hippocampus. iNOS is an enzyme that produces nitric oxide, one of the factors by which post-ischemic oxidative stress contributes to cerebral ischemic damage [18], and edaravone has been shown to reduce iNOS expression following ischemia [19]. As shown in Figure 1, iNOS expression levels were markedly higher in the 2VO group (b) than in the Sham group (a), while 2VO-induced iNOS levels were suppressed in the 2VO + EV group (c). At the same time point, we evaluated the effects of edaravone on ischemic-induced neuronal cell death in the hippocampus. Figure 2A shows that the number of NeuN-positive neurons (red) in the CA1 region was markedly lower in the 2VO group (b) than in the Sham group (a), as we reported previously [14], and also that edaravone suppressed the ischemia-induced loss of neuronal cells (c). Since (1) pyramidal cells in the CA1, 2 and 3 regions were fragile under global cerebral ischemic conditions; (2) delayed neuronal cell death in the CA3 region was observed even eight days after surgery [15,20,21]; and (3) iNOS signals in the CA3 region correlated with neuronal cell damage [22,23], the edaravone treatment used in the present study effectively suppressed the risk of oxidative stress, resulting in successful neuroprotection.

Figure 2A shows the immunoreactivity of phosphorylated CaMK II (p-CaMK II; green). CaMK II is a multifunctional serine/threonine-specific protein kinase in neuronal tissues, and its activity in the hippocampus has been shown to decrease following transient brain ischemia [24], the extent of which has been correlated with the extent of neuronal damage after ischemia [25]. The autophosphorylation of CaMK II was previously demonstrated to be persistently active [26], namely, the activation (=phosphorylation) of CaMK II was crucial for maintaining LTP. In the mouse hippocampus, the immunoreactivity of p-CaMK II was detected in the pyramidal cell layer of the CA1 region, along mossy fibers in the CA3 region, and in granular cells of the dentate gyrus (Figure 2A-d); signals in the 2VO group were very weak in the CA1 ~ CA3 ~ dentate gyrus regions (Figure 2A-e) while those in the 2VO + EV group had clearly recovered (Figure 2A-f). NeuN-positive neurons (Figure 2A-a,c) and p-CaMK II-positive cells (Figure 2A-d,f) had merged (Figure 2A-g,i), indicating that the edaravone treatment significantly restored ischemia-induced decreases in the neuronal network in the hippocampus. 

We determined whether the edaravone treatment affected neurogenesis in ischemic brains. Figure 2B shows the immunoreactivity of DCX, a marker of neuronal precursor cells, in the dentate gyrus subgranular zone (SGZ). The number of DCX-positive cells (green) was very low in the Sham group (a) and 2VO group (b), but was markedly higher in the 2VO + EV group (c). These results suggested that the edaravone treatment accelerated neurogenesis in the SGZ of the ischemic brain.

**Figure 3:**
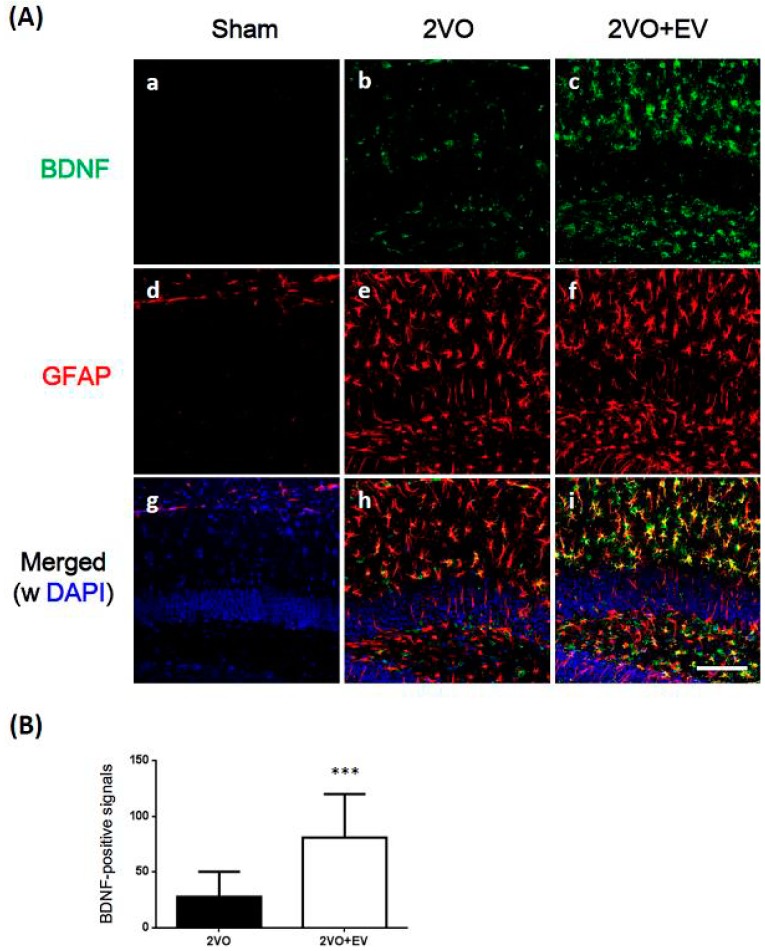
Effects of edaravone on the expression of the immunoreactivity of BDNF and GFAP in the mouse hippocampus. (**A**) Sagittal sections three days after 2VO surgery were stained with an anti-BDNF antibody (green) or anti-GFAP antibody (red), and nuclei were stained with DAPI (blue). Representative immunohistochemical stainings were shown from 18 images per each group. The scale bar shows 100 µm; (**B**) BDNF-positive signals were quantified in three sections per mouse. Values are means ± SD. Symbols show significant differences for the following conditions; 2VO *vs*. 2VO+EV, *** *p* < 0.001.

BDNF has been shown to increase CaMK II activity [27] and stimulates neurogenesis in the SGZ following global ischemia [28,29]. Therefore, we investigated whether edaravone induced BDNF in the hippocampus of these model mice following ischemic surgery. We first tried to detect BDNF protein in the hippocampus by Western blot analysis, but its expression level was too weak to analyze. We then adopted immunohistochemical method to analyze the expression level of BDNF among groups, and it has an availability to identify the cell type of expression. Figure 3A shows that BDNF immunoreactivity (green) was minimal in the dentate gyrus of the Sham group (a), but was higher in the 2VO group (b), as previously reported [13,14,30]. Similarly, the number of activated astrocytes (GFAP; red) was very low in the Sham group (d), but was markedly higher in the 2VO group (e). In the case of the treatment with edaravone, BDNF immunoreactivity in the hippocampus was further increased in ischemic brains (c), and merged with GFAP signals (i). The intensity of the GFAP signal in the 2VO + EV group (f) was similar to that in the 2VO group (e). As shown in Figure 3B, the values of BDNF immune-positive signal counts were significantly higher in the 2VO + EV group than in the 2VO group (*** *p* < 0.001). These results showed that the edaravone treatment induced the expression of BDNF, which was synthesized in astrocytes.

The transcription factor of BDNF is phosphorylated CREB, and its upstream phosphorylating enzyme is ERK1/2 [12,31]. Thus, we examined the effects of edaravone on the phosphorylation of ERK1/2 and CREB in the hippocampus of the ischemic brain using a Western blot analysis. Although both ERK1 and ERK2 are phosphorylated by same kinase, MAPK kinase, only ERK2 isoform has been suggested to be attributable to BDNF production, neurogenesis, and cognitive function [32,33,34]. Consequently, immunoreactive p-ERK2 (42 kDa) band intensities were normalized by ERK2 and p-CREB (43 kDa) band intensities were normalized by CREB in the densitometric comparison.

**Figure 4:**
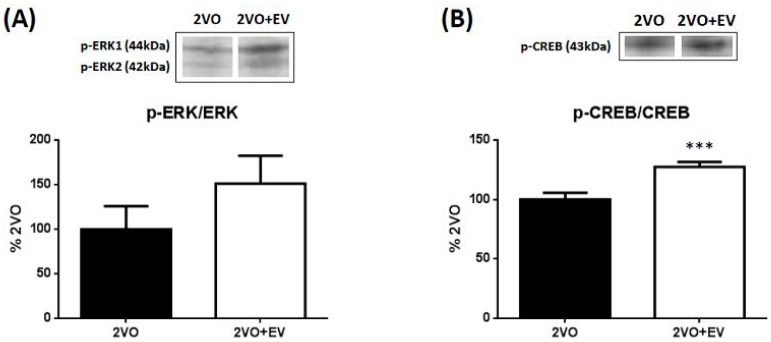
A Western blot analysis of p-ERK2 (**A**) and p-CREB (**B**) in the hippocampus. Hippocampal tissues three days after 2VO surgery were used in a Western blotting analysis. Values are means ± SD. Symbols show significant differences for the following conditions: 2VO *vs*. 2VO+EV, *** *p* < 0.001.

The levels of p-ERK2 and p-CREB in the Sham group were very low; therefore, data in Figure 4 are shown as percentage ratios of the 2VO group. Figure 4A shows that edaravone slightly increased the level of p-ERK2 (*p* = 0.095), whereas Figure 4B shows that edaravone significantly (*** *p* < 0.001) increased the level of p-CREB. These results suggested that edaravone stimulated the synthesis of BDNF in ischemic brains through this cell-signaling cascade.

## 4. Conclusions 

This study shows that edaravone exhibited a novel neuroprotective mechanism of action by accelerating the synthesis of BDNF in ischemic brains through the activation of MAPK signals. The findings indicate that the neuroprotective effects of edaravone are based not only on antioxidant effects but also the enhanced synthesis of BDNF.
